# Friend or Foe? Spontaneous Portosystemic Shunts in Cirrhosis—Current Understanding and Future Prospects

**DOI:** 10.1155/2021/8795115

**Published:** 2021-08-12

**Authors:** Sasidharan Rajesh, Cyriac Abby Philips, Rizwan Ahamed, Jinsha K Abduljaleel, Dinu Chandran Nair, Philip Augustine

**Affiliations:** ^1^Department of GI and HPB Interventional Radiology, The Liver Institute, Center of Excellence in GI Sciences, Rajagiri Hospital, Aluva, Kerala, India; ^2^Department of Clinical and Translational Hepatology, The Liver Institute, Center of Excellence in GI Sciences, Rajagiri Hospital, Aluva, Kerala, India; ^3^Department Gastroenterology and Advanced GI Endoscopy, Center of Excellence in GI Sciences, Rajagiri Hospital, Aluva, Kerala, India

## Abstract

Portal hypertension (PHT) in cirrhosis results from increased resistance to splanchnic blood flow secondary to parenchymal and vascular changes within the liver. In an attempt to counteract the increased portal pressure, two mechanisms simultaneously occur: splanchnic vasodilatation and formation of spontaneous portosystemic shunts (SPSS). Long considered to be a compensatory mechanism to decompress the portal venous system, it is now well established that SPSS are not only inefficient in decreasing the portal pressure but also contribute to reduced hepatocyte perfusion and increased splanchnic blood flow and resistance, associated with worsening PHT. Recent studies have described a high prevalence of SPSS in cirrhosis patients, increasing with liver dysfunction, and observed an association between the presence of SPSS and worse clinical outcomes. In cirrhosis patients with preserved liver functions, the presence of SPSS independently increases the risk of hepatic encephalopathy, variceal bleeding, and ascites, and reduces transplant-free survival. Moreover, the presence of SPSS in patients undergoing transjugular intrahepatic portosystemic shunting and liver transplant has been shown to variably affect the postprocedural outcome. This article provides an overview of the current understanding of the role of SPSS in the natural history of liver cirrhosis and their status as a therapeutic target and an imaging biomarker to identify patients at higher risk of developing complications of PHT.

## 1. Introduction

Portal hypertension, defined as an increase in portal pressure, occurs as a result of angio-architectural changes in liver cirrhosis. Portal hypertension is responsible for most of potentially life-threatening complications associated with cirrhosis [1–3]. Increased resistance to splanchnic venous flow is the initial factor responsible for the rise in portal pressure. This can lead to formation of an extensive network of portosystemic collaterals that divert a fraction of portal blood to the systemic circulation, bypassing the liver. With progression of portal hypertension, these collaterals can increase in size and form large-caliber vascular channels known as spontaneous portosystemic shunts (SPSS) [4].

Traditionally, SPSSs were considered to be a compensatory mechanism to decompress the portal system, protecting against development of esophageal varices, ascites, and gastrointestinal bleeding (GIB) [5–7]. However, recent studies have shown that SPSSs are a marker of severity of portal hypertension [8]. They are not only inefficient in adequately reducing the portal pressure but can also compromise the hepatic perfusion in later stages, leading to progressive liver dysfunction and various other decompensating events [8–12]. Development of SPSS leads to higher incidence of hepatic encephalopathy (HE), gastroesophageal varices, GIB, ascites, hepatorenal syndrome, and spontaneous bacterial peritonitis. This association is pertinent among stable cirrhosis patients in whom significantly more portal hypertension-related complications during follow-up are notable than patients without SPSS [8].

Easier and widespread access to advanced cross-sectional imaging techniques has allowed prompt identification and accurate characterisation of SPSS [13–17]. In patients with recurrent severe HE and gastric variceal bleeding, these SPSSs can serve as a therapeutic target [18–20], while for patients undergoing procedures that relieve portal hypertension like transjugular intrahepatic portosystemic shunting and liver transplantation, the presence of SPSSs has been shown to variably affect the postprocedure outcome [21–23].

In this focussed review, we intend to comprehensively discuss current understanding of the role of SPSSs in patients with cirrhosis, with special emphasis on clinical and therapeutic aspects.

## 2. Pathophysiology and Hemodynamics of SPSS Formation

Traditionally, the development of portosystemic collaterals was considered to be a mechanical consequence of increased portal pressure resulting in passive opening of pre-existing embryonic channels connecting the portal and systemic venous systems. Accordingly, all therapeutic strategies were classically aimed at decreasing portal hypertension. Recent studies, however, have established that active angiogenesis also plays an important role in the development of these aberrant vessels [24–28]. Seminal work by Fernandez et al. in a murine model demonstrated that the formation of portosystemic collateral vessels is mediated by a vascular endothelial growth factor (VEGF)-dependent angiogenic process that can be markedly inhibited by blockade of the VEGF/VEGF receptor-2 signaling pathway [24]. Stimuli such as hypoxia, oxidative stress, inflammation, and shear stress have been shown to drive VEGF overexpression and increased angiogenesis in the splanchnic territory of portal hypertensive rats and cirrhotic patients. VEGF stimulates nitric oxide (NO) production by endothelial NO synthase and increases vascular permeability, which is responsible for the initial collateralization of the portal system [25–28].

While VEGF plays a predominant role in the initial stages of formation of new blood vessels, platelet-derived growth factor (PDGF) helps in stabilizing the vascular architecture of the nascent vessel. Similarily, placental growth factor (PlGF) has been shown to enhance collateral growth by stimulating endothelial and smooth muscle cell growth. Thus, combination of therapeutic strategies directed at inhibiting angiogenesis may have clinical importance in the treatment of established portal hypertension in chronic liver disease and angiogenesis in liver diseases [28]. However, the efficacy and safety of such therapies in routine clinical practice is currently not confirmed.

From a pathophysiological point of view, the formation of collateral vessels, initially driven by the increased portal pressure, contributes to a decrease in hepatocyte perfusion, tissue hypoxia, and consequently the promotion of neoangiogenesis in the splanchnic circulation. This leads to a progressive amplification of the mechanisms causing and maintaining a hyperdynamic splanchnic circulation state, which in turn are responsible for the main clinical events associated with portal hypertension [29].

Kim and Lee have proposed an “electric circuit” theory for the development of SPSS based on the Ohm's law and deduced treatment options and outcomes based on the same [30]. The authors suggest two variables which are primarily responsible for the recruitment of preformed embryonic channels that eventually lead to the formation of SPSS—an increase in portal venous pressure (PVP) and the decrease in shunt resistance (SR). Normally, in the initial stages, the high SR is associated with negligible flow within the shunt. With progression of cirrhosis and the development of portal hypertension, the pressure gradient within the shunt increases, and when PVP becomes sufficiently high, the flow across the shunt goes above zero, resulting in the formation of SPSS. If the SR decreases, the shunt flow increases as demonstrated in cases of aneurysmal dilations of the vascular channels. As these events across the SPSS progress, the PVP and portal blood flow reduces as a result of the circuit bypass created by the shunts. The therapeutic implications of this model is that there occurs an increase of PVP after SPSS occlusion. Hence, when a shunt embolization is planned, one should be aware that the PVP could increase enough to open new SPSS. In this scenario, portal-pressure-reducing strategies such as optimized beta-blocker use and in select patients with advanced portal hypertension complications such as variceal bleeding, ascites, or hydrothorax, the use of concomitant transjugular intrahepatic portosystemic shunt placement may help ameliorate further portal hypertensive events.

## 3. Types of Shunts and Their Reported Clinical Associations

SPSS can be anatomically divided into left-sided or right-sided (or central) shunts based on their location with respect to the midline or splenoportomesenteric vein confluence. These shunts derive their afferent supply either from the branches of splenic vein, namely left gastric, posterior gastric, or short gastric veins, or directly from the superior or inferior mesenteric veins [31, 32].

**Figure 1:**
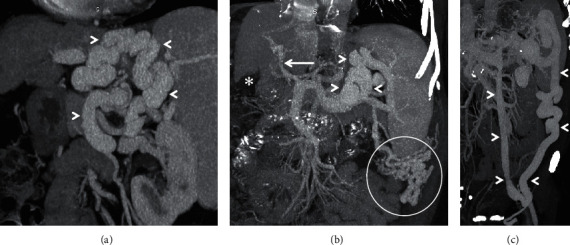
Coronal-oblique MIP images from three different patients depicting splenorenal shunts (arrowheads in (a, b)) and a splenogonadorenal shunt (arrowheads in (c)). *Note.* The presence of additional perisplenic portosystemic collateral vessels in (b) (encircled) with attenuated main portal vein (arrow) and ascites (asterisk).

**Figure 2:**
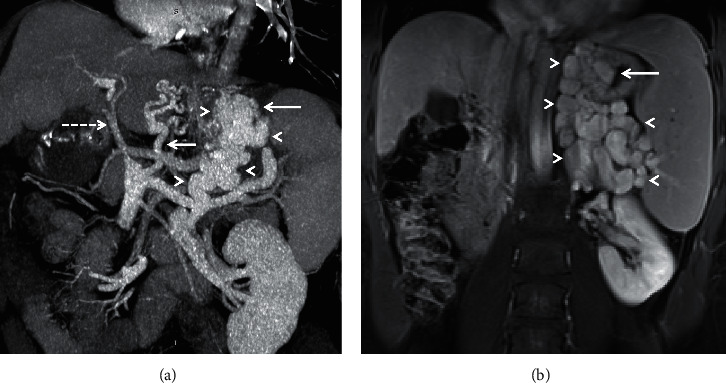
Coronal-oblique MIP (a) and coronal MRI (b) images from two different patients depicting gastrorenal shunts (arrowheads) with varices protruding into the gastric lumen (long solid arrows). *Note.* The attenuated main portal vein (dashed arrow) and prominent left gastric vein (short solid arrow) in (a).

Left-sided SPSS include the splenorenal shunt (SRS), which is one of the most common SPSS identified in patients with liver cirrhosis, the gastrorenal shunt (GRS) and gastrocaval shunt (GCS). SRS is a tortuous, meandering direct communication between splenic vein and left renal vein without intervening the involvement of the gastrointestinal tract (Figure 1). Thus, it is a vascular channel that does not contribute to the formation of varices or risk of spontaneous bleeding. Such a shunt is, in the true sense, a prototype that can lead to portosystemic shunt syndrome (described later) [31, 32]. GRS can be seen in up to 85% of patients with cardiofundal gastric varices (GV) [31]. Although this shunt is a communication between GV and the left renal vein (LRV), in reality, it is a part of the larger portosystemic communication between splenic vein and LRV (Figure 2). Hence, hemodynamically, this is an SRS and should ideally be called the splenogastrorenal shunt [32].

**Figure 3:**
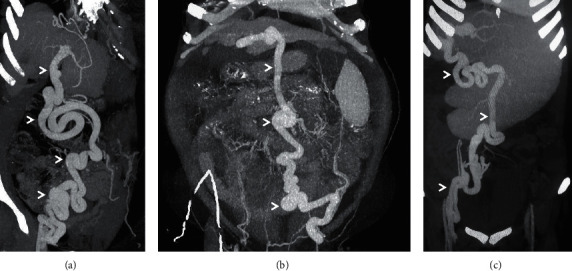
Coronal-oblique maximum intensity projection (MIP) CECT images (a–c) from three different patients depicting the recanalised paraumbilical vein shunt (arrowheads).

**Figure 4:**
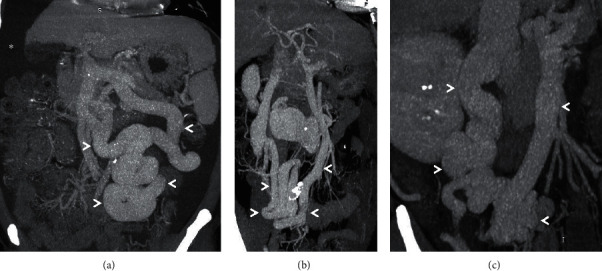
Coronal-oblique MIP images from three different patients (a–c) depicting mesocaval shunts (arrowheads). Afferent vessel for shunt shown in (a) is inferior mesenteric vein while feeder for shunts shown in (b) and (c) is superior mesenteric vein. *Note.* The presence of ascites (asterisk) in (a).

**Figure 5:**
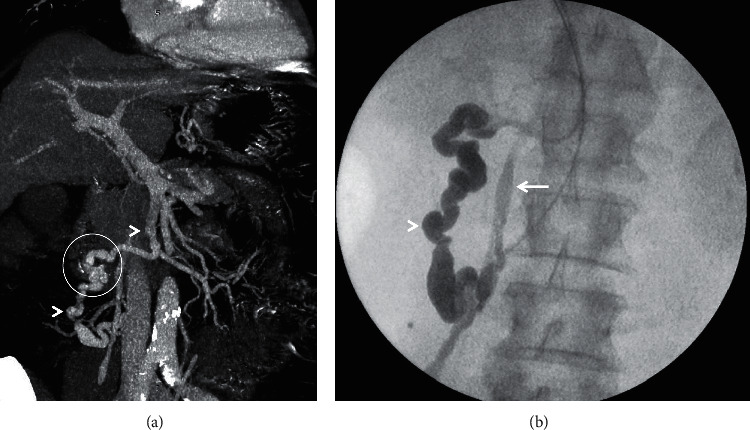
Coronal MIP image (a) depicting a right-sided mesogonadal shunt contributing to ectopic duodenal varices (encircled). Fluoroscopic spot image (b) showing the shunt which was accessed from the transjugular intrahepatic route and occluded with pushable metallic coils and *n*-butyl cyanoacrylate glue (not shown).

**Figure 6:**
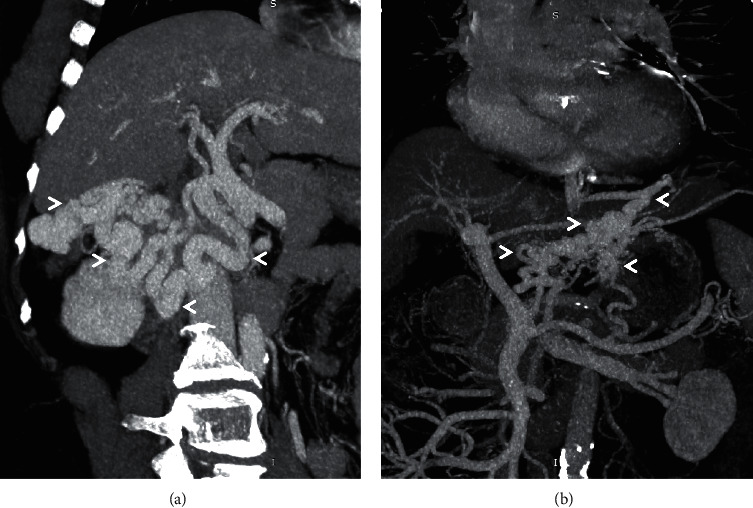
Coronal-oblique MIP images showing a right-sided portorenal shunt (arrowheads in (a)) and a portocaval shunt (arrowheads in (b)).

**Figure 7:**
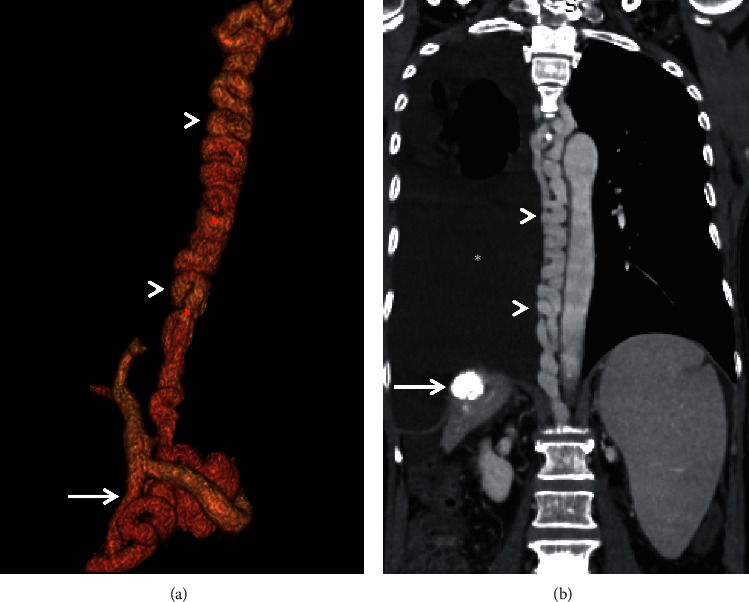
Volume rendered CECT image (a) showing a mesoazygos shunt (arrowheads). Solid arrow shows the superior mesenteric vein. Coronal MIP image (b) of the same patient showing the thoracic portion of the shunt (arrowheads) with massive hepatic hydrothorax (asterisk). Changes of prior transarterial chemoembolization are also noted (solid arrow).

Most common right-sided SPSS is the recanalised paraumbilical vein (RPUV) [4]. RPUV has been associated more with ascites and less with variceal bleeding, while its association with hepatic encephalopathy is controversial (Figure 3). Other uncommon shunts like the mesocaval, mesoazygos, portocaval, portorenal, mesoiliac, and mesorenal shunts, among others, can be either left or right sided (Figures 4–7) [33]. These SPSS have not been studied extensively with regards to their association with various portal hypertensive complications.

### 3.1. Prevalence of SPSS

Advancements in noninvasive imaging techniques have brought about a paradigm shift in the assessment of prevalence of SPSS in patients with liver cirrhosis. Initial postmortem studies and invasive diagnostic techniques like percutaneous transhepatic portography, angiography, and splenoportography have given way to Doppler ultrasound (US), contrast-enhanced computed tomography (CECT), and magnetic resonance imaging (MRI).

Doppler US is a widely available and relatively inexpensive imaging modality that can be performed at bed side and provides useful information about the presence or absence of SPSS and its flow characteristics. In addition, the patency and caliber of portal vein and the direction of flow within it can also be assessed in the same sitting which provides vital clues to the hemodynamic significance of SPSS. However, US is operator dependent and often fails to identify smaller and deeper SPSS due to acoustic interference by the overlying bowel gases. Moreover, accurate delineation of the complete anatomy of SPSS is frequently difficult with US, even in expert hands. CT and MRI, on the other hand, provide a more detailed and global cross-sectional assessment of the entire splenoportal system irrespective of the body habitus of the patient. Considering availability, expense, information provided, and the possibility of performing a three-dimensional reconstruction, CT currently appears to be the most appropriate imaging modality to assess the presence of shunts [34–41].

Earlier studies conducted using Doppler US pegged the prevalence of SPSS between 33% and 42% [42–45]. However, recent studies, performed with CT or MR imaging, point towards a much higher incidence of SPSS in patients with liver cirrhosis [8, 46]. An international multicentre collaborative study conducted by the Baveno VI Cooperation Group found that SPSS were present in 60% of the sample and half of them were classified as large SPSS, with a predefined cutoff of 8 mm [8]. This value was chosen considering the smallest symptomatic embolized shunt reported in the literature. Another retrospective database review reported that 63.5% of patients had SPSS and 18% had a shunt diameter of 1 cm or more [46]. Similarily, a retrospective cohort study of 235 patients found SPSS in 141 patients (60%) [47]. The authors of this study also reported that although the prevalence of SPSS increased with the worsening of liver function or portal hypertension, it remained high (46%–55%) even in the subgroups of patients with compensated cirrhosis, preserved liver function (Model for End-Stage Liver Disease or MELD <10), or liver stiffness measurement <21 kPa, suggesting that SPSS may be commonly found even in early stages of cirrhosis. Similar findings were reported in the study by Baveno VI cooperation group.

With regard to the type of SPSS, majority of studies have found that recanalised paraumbilical vein (RPUV) shunt and splenorenal shunt are the most commonly found SPSS. A significant percentage of patients—between 20% and 25%—had more than one SPSS. Interestingly, splenorenal shunt was the most commonly found large SPSS, while RPUV was the most frequently seen SPSS when the size criterion was not considered [8, 46, 47].

There appears to be some association between the etiology of liver cirrhosis and the presence of SPSS. Several authors found that portosystemic shunts (RPUV, in particular) were more common in patients with alcohol-associated cirrhosis. This was initially attributed to a delayed diagnosis of liver disease in these patients [8, 9]. However, in a recent retrospective cohort study, it was reported that the higher risk of SPSS in nonviral etiology was independent of liver function or portal pressure. The authors attributed this finding to the different patterns of fibrogenesis and severity of portal hypertension described in the various etiologies of liver cirrhosis [47].

### 3.2. Complications Related to SPSS

Studies reporting an association between SPSS and portal hypertensive complications have shown contradictory results. Earlier studies had suggested that the presence of a large SPSS may have a protective effect against the development of esophageal varices (EV) and ascites, especially in patients with HE. In a study by Onishi et al., patients with SPSS and HE had fewer EVs and a reduced incidence of acute variceal bleeding [48]. Takashi et al. also found a lower incidence of EV in patients with SPSS and HE [5]. A case-control study by Riggio et al. reported that patients with chronic HE and large SPSS had lower EV, ascites, and portal hypertensive gastropathy than patients without SPSS, which was supportive of a compensatory mechanism [6]. Similarily, Tarantino et al. showed that patients without SPSSs had a higher rate of large EV [7].

Recent studies, however, have found that patients with large SPSS and HE show more signs of clinically significant portal hypertension in the form of ascites and varices. Berzigottti et al. reported that patients developing new abdominal portosystemic collaterals during follow-up had a significantly higher rate of EV formation compared with patients with unchanged doppler US findings, suggesting that abdominal collaterals are not protective from the formation or growth of EV [42]. The same authors also showed in another study that 90% of patients with cirrhosis and SPSS had hepatic venous pressure gradient (HVPG) higher than 16 mmHg, which was linked to an increased risk of decompensation and death [49]. More recently, Simon Talero et al., in a large international multicentre study, reported that patients with SPSS more often had HE, ascites, variceal bleeding, infections, and acute kidney injury [8]. Interestingly, these differences were significant among those with preserved liver function (MELD score of 6–9 or Child–Pugh class A). Cirrhosis patients with large SPSS had higher Child–Pugh and MELD scores than those with small collaterals. Nonetheless, both had worse liver function than patients without shunts. Nardelli and colleagues found that the presence of SPSS on CT images in patients with cirrhosis was associated with higher mortality and complications, including HE, variceal bleeding, and portal vein thrombosis [46].

These contrasting findings can be explained by the dynamic nature of liver cirrhosis which goes through different stages and is affected by different compensatory mechanisms [50]. In the first functional pathophysiology, SPSS could represent an inefficient compensatory mechanism that partially reduces portal hypertension and its complications by rerouting portal blood away from the liver. As portal hypertension progresses, these SPSSs hypertrophy and the volume of portal blood diverted into the systemic circulation increases. Progressively, the portal vein becomes attenuated or thrombosed and the flow within it becomes hepatofugal, resulting in the SPSS becoming the only outflow of the splanchnic circulation. Earlier studies on this subject were cross-sectional and retrospective in nature which might have led to a different interpretation of the results.

**Figure 8:**
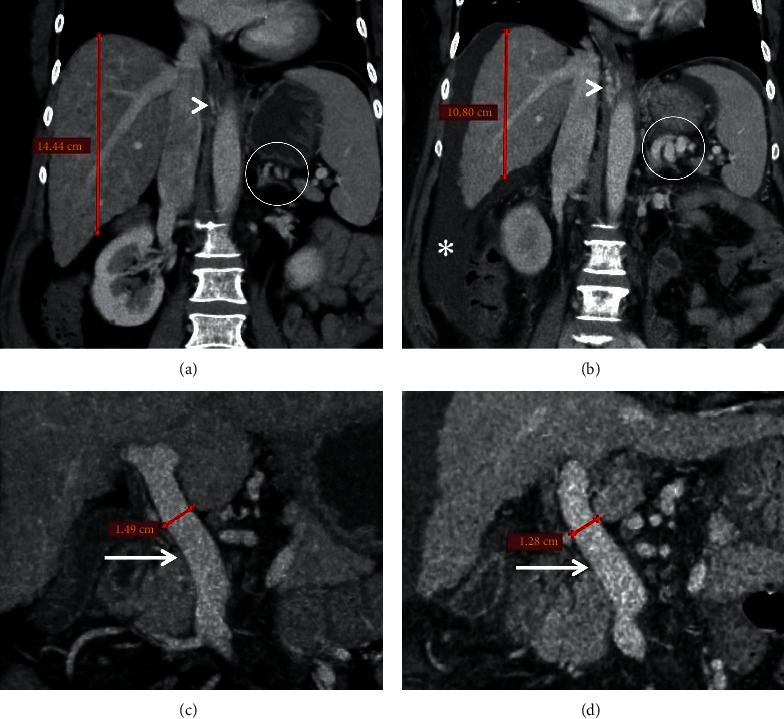
Coronal (a, b) and axial (c, d) CECT images of a patient taken 1 year apart demonstrate atrophy of liver (calipers in (a, b)), decrease in the caliber of main portal vein (arrows in (c, d)), interval development of ascites (asterisk in (b)), and esophageal and paraesophageal collaterals (arrowheads) with enlargement of the splenorenal shunt (encircled).

It follows that the development of SPSS has implications on liver function. Kumamoto et al. proposed the term “portosystemic shunt syndrome” which is characterized by the deterioration of liver functions in the form of worsening Child–Pugh scores over 5 years, as compared with patients with cirrhosis and portal hypertension without gastrorenal shunts [10]. Accordingly, Saad et al. described a complete syndrome with clinical manifestations and imaging findings that develops in three phases: (1) early stage, characterized by infrequent HE episodes, no ascites, and well-preserved liver function; (2) late stage, in which, HE episodes occur more frequently along with decline in liver function; radiological signs include reduced liver volume, sluggish portal flow, diminutive portal vein branches, and high risk of portal thrombosis; and (3) end stage, in which, HE is persistent with overt episodes, and the patient has advanced liver failure with portal vein thrombosis (PVT) (Figure 8). The amount of portosystemic shunting that is significant is variable from one patient to another depending on the degree of underlying liver disease, location of the shunt, peripheral resistance of the portal circulation and the shunt itself, the presence of other SPSS and medical intervention and its response and optimization such as beta blockers and diuretic use [11].

It is important to note that PVT can be both a cause and effect of portosystemic collateralization. Just like large SPSS can lead to PVT, the reverse is also true. Portomesenteric vein thrombosis due to any other cause can lead to the formation of portosystemic collaterals. As a general rule, to differentiate between cause and effect, PVT leading to shunt formation has variceal dominance, with preponderance of varices and numerous portoportal or portosystemic shunts or both. On the contrary, the predominant morphologic feature of PVT caused by SPSS is the paucity of varices. Clinically, the predominant presentation of shunts caused by PVT is variceal bleeding, whereas the predominant clinical presentation of large SPSS causing PVT is a history of recurrent or refractory hepatic encephalopathy and hypersplenism [11].

#### 3.2.1. Hepatic Encephalopathy

Ineffective liver detoxification due to rerouting of portal blood through low-resistance SPSS and hepatic impairment due to decreased liver perfusion leads to hepatic encephalopathy (HE), in which accumulation of inflammatory and neurotoxic components result in psychomotor and cognitive disturbances. The association between HE and the presence of SPSS is well known and documented in literature. Studies show that 46% to 71% of patients with recurrent or persistent HE had the presence of large SPSS on imaging. Riggio et al., in a case-control study, found large SPSS in 71% of patients with chronic HE, while only 14% of the group without HE had SPSS [6]. A relationship between SPSS size and HE has also been observed as demonstrated by Praktiknjo et al., in which, large SPSS, classified according to the total shunt area, had higher risk of developing HE and higher ammonia levels [51].

Interestingly, patients with cirrhosis and SPSS can develop HE in the presence of stable liver functions and absence of identifiable precipitating factors. Therefore, in the setting of recurrent or persistent episodes of HE in a patient with relatively preserved liver functions, the presence of large SPSS should be actively sought. CT is the preferred imaging modality in such situations, as it can identify and precisely delineate the anatomy of SPSS, some of which (especially the deeper and more centrally located ones) can be missed on doppler US. Moreover, a high rate of minimal HE has recently be reported in cirrhosis patients with large SPSS, which was further associated with a significant risk of developing overt HE on follow-up [52].

Cirrhosis patients with recurrent or persistent HE can also develop a bradykinetic-rigidity syndrome referred to as “cirrhosis-related Parkinsonism” which is characterized by ataxia, dystonia, choreoathetosis, or spastic paraparesis and a slow progressive decline in cognitive dysfunction. Although rare, this difficult-to-treat form of HE is frequently noted in the presence of large SPSS. Hepatic myelopathy, another rare but disabling form of HE characterized by progressive spastic paraparesis and hyper-reflexia was shown to be associated, in up to 85% of cases, with large SPSSs [31].

Patients with large SPSS may benefit from better tailored and optimized antiammonia measures along with education and awareness on precipitating events such as constipation, use of sedative drugs, diuretic treatment overuse, and early identification and treatment of infections. Even in the wake of optimization, recurrent or persistent HE occurs and then interventional management should be offered early in the course of the disease.

Large shunts, defined as those of diameter ≥8 mm, can be embolized through a variety of percutaneous endovascular techniques. Among carefully selected patients, embolizing shunts (Table 1) to treat recurrent or refractory HE was found to be both efficient and safe [18, 53–58]. After embolization, at 3 months, around 60% of patients and a high percentage remain free of HE at 1 to 2 years (49%–55%), respectively. Late recurrences of HE due to the development of new collaterals or recanalization of previously occluded shunts notably occur in a small proportion of patients, especially those with high MELD score at baseline. Initial embolization procedures were done exclusively by balloon-assisted retrograde transvenous occlusion (BRTO) technique. Transfemoral or transjugular approach-based BRTO occludes the shunt outflow via use of an occlusion balloon, followed by injection of a sclerosant mixture such as sodium tetradecyl sulfate foam with lipiodol or gelfoam slurry. The indwelling balloon mainly acts as the hemostatic unit within the shunt and also prevents sclerosant back-leak into the systemic circulation. In BRTO, the balloon has to be kept inflated within the shunt from 6 hours to sometimes up to 20 hours and is removed only after the stagnation of sclerosant is confirmed on imaging. The need for continuous monitoring, long procedure timing, instances of balloon rupture and sclerosant embolization are some of the major concerns associated with BRTO. This has led to several shunt embolization technical modifications aimed at improving patient safety and logistics. These include coil-assisted and plug-assisted retrograde transvenous obliteration (CARTO and PARTO, respectively) in which, essentially, the role of balloon is taken over by coils or plug. These coils or plugs need not be removed and can be left behind as permanent embolising agents, thus, reducing the procedure time and reducing the risk of complications [31].

**Figure 9:**
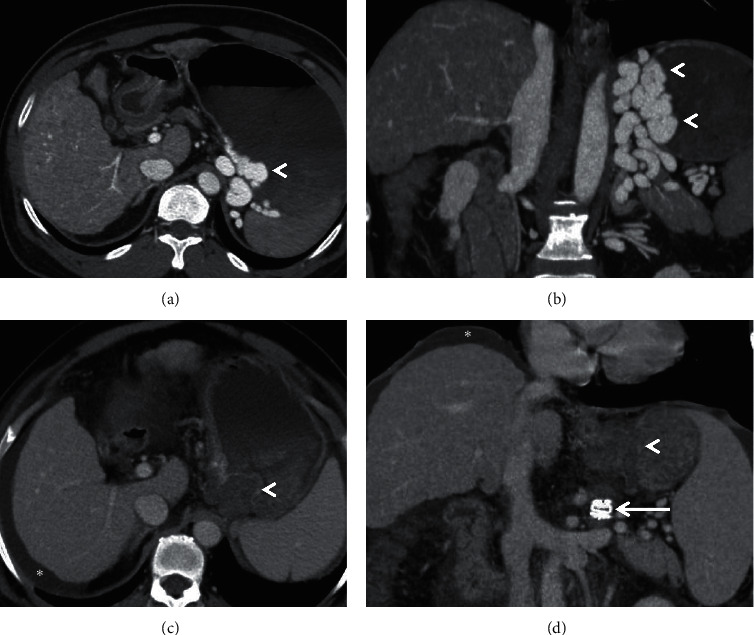
Axial (a, c) and coronal (b, d) CECT images depicting gastric varices (arrowheads in (a, b)) associated with a gastrorenal shunt in a 58-year-old patient with liver cirrhosis and intractable gastric variceal bleeding. The patient underwent plug-assisted retrograde transvenous obliteration (PARTO) of the shunt and variceal complex. Postprocedure images (c, d) show completely thrombosed varices (arrowheads) with vascular plug-in-situ (arrow). *Note.* The interval appearance of mild ascites (asterisk) after the procedure which responded to diuretics.

Studies have shown that blood flow within the portal vein significantly increases at 1 and 12 weeks after shunt embolization in cirrhosis patients with Child–Pugh A and B status [59]. In addition, improvements in liver function, reflected by increase in serum albumin levels in the absence of protein supplementation were notable. However, procedural complications due to worsening of portal hypertension have also been reported in a small subset of patients [60]. New onset or worsening ascites in approximately 30% of patients which usually respond to diuretic therapy is also noticed after shunt occlusion (Figure 9). Life-threatening uncontrolled acute esophageal variceal bleeding during the follow-up period after shunt embolization is another complication that requires endoscopic surveillance. Thus, careful selection of patients for shunt embolization procedure is of paramount importance. Patients with recurrent or refractory ascites or large gastroesophageal varices are not ideal candidates for shunt occlusion. The MELD score pre-embolization was identified as a good predictor of outcomes, with a range of cutoffs from 11 to 15. Similarily, the largest single-centre study on shunt embolization from India showed that Child–Pugh score >11 predicted mortality postshunt occlusion, and hence, such patients need to be excluded from shunt embolization for recurrent or persistent HE and be listed for liver transplantation as the treatment of choice [58]. In the study by Ishikawa et al., low liver stiffness values with the cutoff 21.6 kPa (correlating with clinically significant portal hypertension) measured by transient elastography, were linked to better outcomes [61].

The increased risk of complications of shunt embolization in patients with advanced liver disease makes early identification of SPSSs and prompt intervention in these patients an attractive alternative. In a recent retrospective study of 45 patients, Philips et al. evaluated the utility of early (after the first episode of spontaneous shunt-related overt HE) versus late (in SPSS-related recurrent or refractory HE) shunt embolization of large PSS in patients with cirrhosis and HE [62]. The authors found that early shunt embolization compared with no or late embolization leads to better reduction in portal hypertension events, lesser frequency of portal vein thrombosis, and improved disease status and survival. The authors hypothesized that management of PSS in cirrhosis early in the course of the disease may help change the natural course of the disease. However, larger prospective trials on the timing of shunt occlusion are needed.

#### 3.2.2. Gastric Variceal Bleeding

Gastric varices (GV) are seen in 5% to 33% of patients with cirrhosis and portal hypertension [63, 64]. Bleeding from GVs occur less frequently than esophageal varices (GV vs EV, 10%–30% only), but the severity of bleeding is often higher with increased requirement for blood transfusions, higher rates of failure to control bleeding, early rebleeding and recurrent bleeding (more for GOV2 and IGV1; cardiofundal varices) with mortality rates reaching up to 20%. This is because cardiofundal varices are associated with large gastrorenal shunts (GRS) in up to 85% of cases and have a “downhill” drainage as opposed to an “uphill” drainage of EV *via* azygos-hemiazygos venous system [65]. The GRS allow for partial decompression of the portal venous system while carrying large amounts of venous blood within it. Consequently, GV exist as “low pressure, high volume” channels and can bleed at lower pressures than esophageal varices (15–20 mm Hg vs 21–23 mm Hg, respectively) [66, 67]. More importantly, between 10% and 16% of gastric varices can bleed at portosystemic gradient (PSG) <12 mm Hg [64]. Thus, the management of GV hemorrhage (GVH) requires a different therapeutic approach, and the optimal treatment algorithm inclusive of portosystemic shunt occlusion still remains to be established.

**Figure 10:**
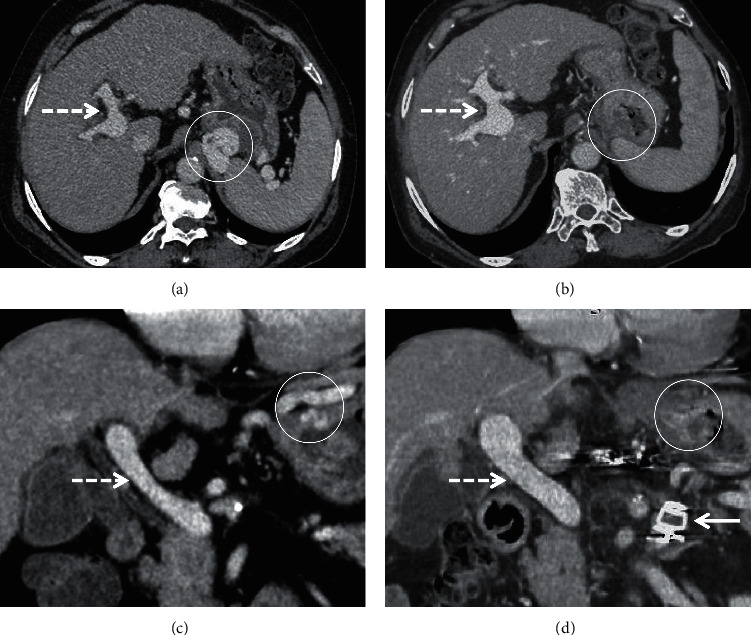
Fluoroscopic spot images depicting persistence of a splenorenal shunt (arrowhead in (a)) after placement of a TIPS stent (dashed arrow). The shunt was occluded by combined antegrade-retrograde accelerated trap obliteration technique using vascular plugs (solid arrows) and sclerosant mixture.

Percutaneous endovascular therapy is indicated for GV bleeding that is nonresponsive to medical and endoscopic management transjugular intrahepatic portosystemic shunt placement can help attain hemostatic control in up to 90% of cases of acute GV bleeding. However, it is not proven to be as efficacious in this setting as in bleeding from esophageal varices. This is because GVs can bleed at lower PSG than EV. Studies have shown that approximately 25% to 30% of GVs can persist and rebleed after successful TIPS placement [68]. Certain theories have been proposed for the suboptimal efficacy of TIPS in controlling GVH. These include the “proximity”, “throughput,” and “recruitment” theories [68–70]. The “proximity theory” suggests that GVs (supplied more commonly by posterior and short gastric veins) are anatomically farther away from the TIPS stent, and hence less likely to be decompressed compared with EVs which are supplied predominantly by the left gastric vein. The “throughput theory” states that SPSS associated with GVs can compete with the TIPS stent leading to early TIPS dysfunction. Finally, as per the “recruitment theory”, new feeder-collaterals develop after proximal embolization of a GV complex leading to persistence of varices and further bleeding risk. These factors have led to the development of obliterative therapies, like BRTO, or its modifications such as plug-assisted (PARTO) or coil-assisted transvenous occlusion (CARTO) in the management of GVH. These therapies are aimed at controlling both inflow and the outflow of the variceal complex using balloon, coils, or plug. Various studies and subsequent meta-analyses (Table 2) have reported technical and clinical success rates in excess of 95% for BRTO [71–82]. Also, gastric variceal rebleed rates among those undergoing successful BRTO procedure range between 0% and 20% [71–75, 79–82]. Compared to TIPS, shunt embolization results in diversion of blood towards the liver, thereby preserving or improving liver functions, during the initial 6 to 9 months [71, 73, 75]. In addition, BRTO is efficacious in patients with recurrent shunt-related hepatic encephalopathy, unresponsive to medical therapy. Thus, patients who are at high risk of developing HE after TIPS can also safely undergo BRTO. However, occlusion of GRS can aggravate sequelae of portal hypertension because these constitute portosystemic shunts that decompress the portal venous system (Figure 10). Long-term follow-up of patients who underwent BRTO have shown development of esophageal and duodenal varices, ascites, hydrothorax, and portal hypertensive gastropathy. Prospective studies and meta-analysis comparing TIPS and BRTO in the management of GV have found that the latter is at least as efficacious as the former in controlling the acute episode of hemorrhage with a trend towards lower incidence of rebleeding [75–78]. Of note, BRTO was associated with lower postprocedure HE and mortality at 1 year [76]. A very recent study was the first randomized controlled trial to demonstrate the superiority of BRTO in the management of (re)bleeding from GVs. In this study, Luo and colleagues found that BRTO as the therapeutic modality for bleeding GVs resulted in fewer hospitalizations, in-patient stays, and lower medical costs [83].

**Figure 11:**
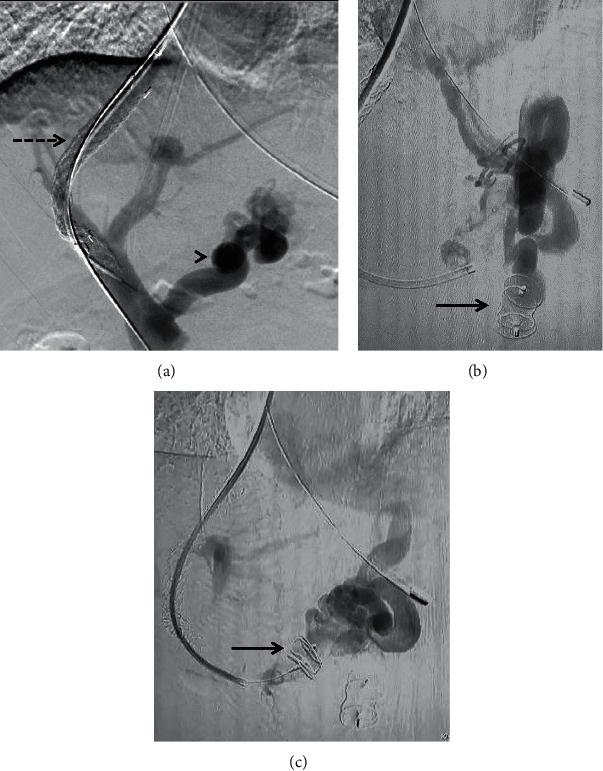
Axial (a, b) and coronal (c, d) CECT images depicting gastric varices (encircled in (a, b) in a 74-year-old patient with liver cirrhosis and intractable gastric variceal bleeding. Post-PARTO images (c, d) show completely thrombosed varices (encircled in (c, d)) with vascular plug-in-situ (arrow in (d)). *Note.* The interval increase in the caliber of main portal vein and its intrahepatic branches (dashed arrows).

Lately, a combination of TIPS and BRTO has been used for the management of GV [65, 79, 84]. Since obliteration of GRS can lead to worsening of portal hypertension, simultaneous or staged placement of TIPS can ameliorate these symptoms. Combining TIPS with shunt embolization helps ameliorate the risk of development of HE because GRS are often larger in diameter and have higher flow rates compared to TIPS. Furthermore, occlusion of the competing GRS reduces the chances of TIPS dysfunction in the long term. Typically, TIPS is performed first and a splenoportogram is obtained. The GRS is cannulated retrogradely from the left renal vein and suitable sized vascular plug or coils deployed. The inflow vein is then occluded with a balloon or plug and sclerosant injected into the variceal complex to achieve complete obliteration. This procedure is called combined antegrade-retrograde accelerated trap obliteration (CARATO, Figure 11) [65]. It allows clear delineation of the complex GV inflow anatomy, GV inflow and outflow control, and avoidance of the need for prolonged balloon inflation, as in traditional B-RTO. Performing BRTO followed by TIPS improves technical success in cases where the portal vein is severely attenuated due to the siphoning of blood away from the liver by the large GRS [65, 66]. These diminutive portal veins can be difficult to target during TIPS and may require additional techniques for achieving the desired results. Following BRTO, due to the diversion of blood towards the liver, caliber of portal vein may improve making it an easier target.

### 3.3. Influence of SPSS on Outcome of TIPS Procedure

Transjugular intrahepatic portosystemic shunting (TIPS) has become an established treatment option for complications of portal hypertension such as acute or recurrent variceal bleeding and difficult-to-treat ascites [85]. TIPS is minimally invasive and achieves impressive reduction in portal pressure. Nevertheless, increased incidence of HE and risk of hepatic dysfunction due to diversion of portal blood flow remain significant issues with TIPS.

**Figure 12:**
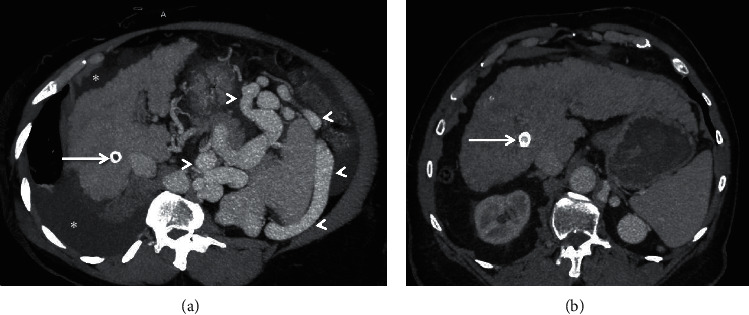
Axial CECT images depicting a large and tortuous splenorenal shunt (SRS; arrowheads) with thrombosed TIPS stent (arrow in (a)). In addition, the image shows recurrence of ascites and hydrothorax (asterisk) for which TIPS was done. The patient underwent occlusion of the SRS and revision of TIPS stent. The postprocedure image shows patent TIPS stent (arrow in (b)) with nonvisualization of SRS and the absence of ascites.

Large SPSS can often be found at the time of splenoportography in patients undergoing TIPS. Logically, the pre-existing SPSS should collapse after placement of TIPS stent due to the normalization of portal pressure and resultant decrease in blood flow in these aberrant vessels. However, it has been shown that even after TIPS placement, nearly one-third of SPSSs remain unchanged and can potentially compete with TIPS for portal flow (throughput theory) (Figure 12) [21]. These shunts, especially when associated with varices, can lead to increased incidence of rebleeding. Furthermore, the placement of TIPS stent in such patients has been shown to increase the risk of HE because TIPS would result in additional portosytemic shunting and decrease the already compromised hepatic portal perfusion. Nevertheless, it is unclear whether a coexistent SPSS has an impact on post-TIPS outcomes and more importantly, whether they need to be embolized. Occluding them during TIPS might decrease the incidence of HE and rebleeding rates and improve hepatic synthetic function but may also theoretically lead to aggravation of portal hypertension.

He and colleagues in a retrospective study found that a pre-existing large nonvariceal SPSS was associated with a higher risk of overt HE, which was decreased by prophylactic SPSS embolization during TIPS [86]. Moreover, embolization had no clear influence on clinical relapse, shunt dysfunction, and mortality after TIPS. Similar results were obtained by Leng et al. who evaluated combination of TIPS and shunt embolization in variceal bleeding [87]. Another recent retrospective single-centre study of 40 patients compared the safety and clinical outcomes of combined TIPS and variceal obliteration to those of TIPS alone for the treatment of GV [88]. The authors found that GV eradication rate is significantly higher after combined therapy, with no associated increase in portal hypertensive complications.

Based on current evidence, it appears prudent to embolise shunt of any size contributing to the formation of varices during TIPS. For nonvariceal SPSS, a decision can be taken based on the size of shunt and post-TIPS splenoportogram. Any large SPSS (defined as >8 mm in caliber) or shunt of any size which shows contrast opacification on completing splenoportogram should ideally be embolised to decrease the risk of post-TIPS HE, liver failure, and early TIPS dysfunction.

### 3.4. Influence of SPSS on Outcome of Liver Transplantation

After orthotopic liver transplantation (LT), portosystemic collaterals typically collapse, but large SPSSs—specifically those more than 10 mm in diameter—are less likely to involute and may continue to steal flow from the liver bed. Multiple published studies have reported that large SPSSs are associated with increased rate of complications after LT. These include primary nonfunction and dysfunction of the graft, higher risk of portal vein thrombosis, and reappearance of HE after LT [89–107]. These complications are thought to be driven by the diminished perfusion of the graft, in the presence of persistent shunt flow. In addition, shunts may reconstitute, and the steel may worsen if the intrinsic allograft vascular resistance becomes elevated, as may occur with graft rejection, fluid overload, and other posttransplant complications [107–110]. Thereby, SPSS ligation during LT has been proposed and successful short-term outcomes reported [99–101, 105–107]. Some studies advocate preoperative percutaneous endovascular embolization of SPSS if the Doppler US assessment shows sluggish hepatofugal flow within an attenuated portal vein. Some other groups recommend that the portal flow be assessed intraoperatively and decision to intervene be made based on the evidence for inadequate flow to the allograft. However, there are concerns about procedure-related complications, such as bleeding or inferior vena cava thrombosis [100, 102, 103]. Moreover, it remains controversial as to whether the persistence of SPSS and portal steal is uniformly detrimental to long-term allograft function. Therefore, many centres follow the practice of close monitoring of the SPSS and functional status of the transplanted liver and intervene only if the SPSS becomes symptomatic [106].

Gómez-Gavara et al. conducted a retrospective study on 66 consecutive patients with SPSS >1 cm who underwent LT. Based on the effect of SPSS clamping/unclamping test on flow within the portal vein during the anhepatic phase, approximately half of these patients had the shunt ligated during the surgery [107]. The authors found that SPSS ligation during LT was associated not only with lower postoperative morbidity, HE and PVT, but also with better patient and graft long-term survival during a mean follow-up of 25 months. However, primary graft nonfunction/dysfunction rates did not differ significantly between the two groups suggesting that early graft function was not affected by the intervention. In addition, the authors advocated against shunt ligation in patients with small-sized graft and when ligation of SPSS is difficult from a technical point of view.

Recently, Allard et al. observed that PVT and SRS size in recipients of living-donor LT were independent predictors of postoperative portal complications. These complications included portal vein stenosis or thrombosis requiring surgical, percutaneous, or medical management [108]. The observed risk among recipients with pre‐LT PVT was 8.3% when the SRS was ≤7 mm, increasing to 38.5% when the SRS was >15 mm. The authors thereby proposed consideration for intraoperative intervention in cases with a large SPSS and pre-LT PVT.

However, an association between SPSSs and post-LT complications has not been observed by all groups. Saks et al. in their retrospective study found that 23% of patients had an SPSS while 77% did not [109]. In the presence of SPSS, patients were more likely to have a PVT and gastroesophageal varices on imaging and less likely to have ascites. Even in the absence of shunt ligation, almost half of the evaluated shunts spontaneously decreased in size after LT. Nonligated large SPSSs were not associated with increased risks for mortality or graft failure after LT. However, this study did not methodically evaluate subgroups of SPSS patients who were at higher risk for an adverse clinical course such as those with impaired portal vein inflow or risk factors for persistent hepatic encephalopathy in the post-LT period. Similarly, Rodrígez et al. did a retrospective study of 326 patients, out of which 113 had large SPSS (defined as >8 mm in diameter) and 150 had small SPSS [110]. Only five large SPSSs from a cohort of 263 shunts were ligated during LT. The authors found that SPSSs did not influence mortality or graft survival, regardless of the size of the collateral and the type of graft used.

To summarize, the management of SPSSs in LT remains controversial with current recommendations suggesting ligation of SPSSs in high-risk patients with low portal venous flow or PVT or in those with large shunts (>8–10 mm in diameter), to avoid HE, graft hypoperfusion and otherportal complications. It is prudent to not ligate SPSSs in patients with small-sized grafts and technically difficult situations. On follow-up, in the presence of new onset portal system related complications or graft dysfunction due to the persistence of symptomatic large SPSSs after LT, shunt embolization could be considered on a case basis.

### 3.5. SPSS as a Prognostic Marker

**Figure 13:**
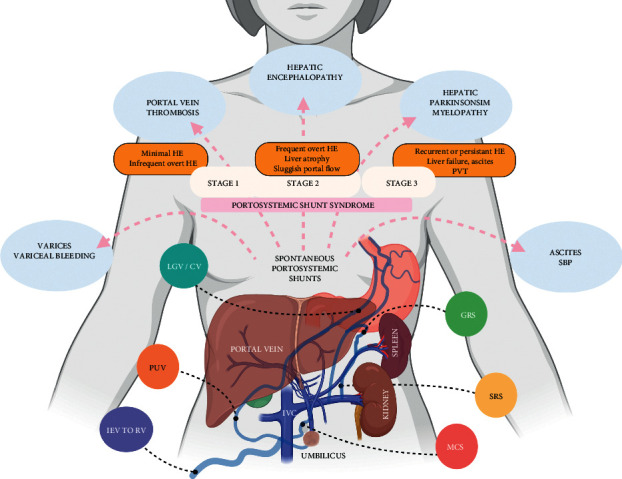
Schematic infographics showing the stages and impact of large spontaneous portosystemic shunts in patients with cirrhosis.

In a study conducted by the Baveno VI Cooperation Group, authors found that SPSSs were independently associated with mortality or LT [8]. This was more appreciable in the group with preserved liver function (MELD score of 6–9). The authors did not find any relationship between mortality and SPSS size or anatomical type. However, Praktiknjo et al. recently used the sum of the cross-sectional areas of all SPSSs identified, reporting that a large SPSS area (>83 mm^2^) was associated with worse survival [51]. In addition, another recent retrospective cohort study of 235 advanced chronic liver disease patients found that the presence, size, and number of SPSS predicts the risk of decompensation across all stages of cirrhosis [47]. SPSS presence was associated with a 2.3-fold increase in the risk of any event of decompensation. The best shunt diameter cutoff to predict the development of decompensation was 8 mm. This result remained significant across all the prognostic stages of cirrhosis (D'Amico staging) and independent from the history of decompensation and the presence of high-risk varices. The authors also found that the presence of gastrorenal shunts was consistently associated with an increased risk of decompensation and was an independent predictor of transplantation or liver-related death, suggesting that not all SPSS are the same in terms of prognostic significance (Figure 13). Yi et al. found that cirrhotics with large SPSS had significantly thinner diameters of main and right branch of portal vein compared to those without. The severity of liver disease was higher in those with SPSS with more reduction in liver volume, higher liver function impairment and ultimately, increased mortality [111].

These results suggest the relevance of identifying and characterizing (numbers, size, and location) SPSS, specially the subgroup of cirrhotic patients with preserved liver function in whom the presence of SPSS could serve as an imaging biomarker to predict higher risk of complications and lower survival. These patients would probably benefit from a closer surveillance and more intensive therapy. Yet, no universal or validated protocol for SPSS detection and especially reporting is available today. The role of artificial intelligence or other (semi)automated software-based algorithms for comprehensive prediction of SPSS, based on clinical or investigational parameters with or without imaging may be a valuable diagnostic tool of the future.

## 4. Conclusion

Large SPSS are not compensatory mechanisms to decompress the portal system in patients with cirrhosis. On the contrary, they serve as markers of severe portal hypertension and are independently associated with complications such as ascites, HE, portal vein thrombosis, and progressive liver failure that occur early in the natural history of cirrhosis which portend worse outcomes. The beneficial role of early management of SPSS is notable in cirrhosis patients who present with variceal bleeding or recurrent HE. However, the role of primary management of SPSS and impact on the natural history of cirrhosis remain enigmatic. Combined approaches of shunt embolization and amelioration of portal hypertension *via* TIPS placement in selected patients may help improve clinical outcomes and pending further quality prospective studies. The presence of SPSS can serve as an imaging biomarker to identify the subset of patients with liver cirrhosis but preserved hepatic functions in whom, early and severe portal hypertensive events may complicate the natural history of disease, which may benefit with early aggressive therapeutic interventions to prolong life. Cirrhosis patients with SPSS and associated clinical events need closer surveillance and more intensive therapeutic options that include the need for early interventional management of shunts, pending further high-quality studies. Future directions in portosystemic shunt syndrome include identifying the role of antiangiogenic treatment as a therapeutic target to prevent the formation of portosystemic collateral pathways and shunts and of imaging surveillance to detect the formation of new SPSS. Further studies on the effect of size and type of shunt on the natural history of liver cirrhosis and definitive role of early shunt embolization in selected group of patients remain an unmet need.

## Figures and Tables

**Table 1:** Overview of recent studies on efficacy and safety of portosystemic shunt embolization for recurrent hepatic encephalopthy.

Authors, year	*n*	Types of shunt	Technique; success rate	Follow-up	Complications	Efficacy; comments
Mukund et al, 2012	7	Splenorenal-7	BRTO; 86%	4 months (mean)	2 early complications (hepatic and renal dysfunction with bacteremia)-responded to medical management	HE improvement: 100%; retrospective, single-centre study, small sample size, limited follow-up; no endoscopic or long-term imaging data
Laleman et al, 2013	37	Splenorenal-20 Paraumbilical-9 Mesocaval-7 Mesorenal 1	CARTO, PARTO; 100%	697 days (mean)	8 early complications-7 mild and 1 capsular bleed De novo EV-2, EV bleeding-1, nonfatal Ascites-no significant difference PVT: 4 (11%; 1 in PV, 3 in one branch)	HE improvement: short-term (100 days): 59.4% Long-term (2 years): 48.6%; Retrospective, multicentre study
Lynn et al, 2013	20	Splenorenal-12 Rest-other types	CARTO, PARTO; 100%	12 months (median)	2 early complications-1 mild, 1 cholangitis De novo EV-1 Ascites-6 (4 requiring paracentesis)	HE improvement: Short-term (1–4 months): 100% Long-term (6–12 months): 92%
An et al, 2014	17	Splenorenal-14 Paraumbilical– 3	CARTO, PARTO; 100%	19 months (median)	No procedure-related complications Ascites–3 (mild) EV: 3, no GIB, no PVT	Recurrence of HE for 2 years: 39.9% (embolized) versus 79.9% (control)
Naeshiro et al, 2014	14	Splenorenal-3 Gastrorenal-4 Mesocaval-5 Portocaval-2	BRTO, CARTO; 92.9%	27 months (median)	No serious procedure-related complications EV: worsening at 3 months (21%), worsening at 24 months (29%) GIB: 14%	HE disappearance in 1–2 weeks: 93%
Inoue et al, 2014	19	Splenorenal-19	BRTO; 100%	28 months (mean)	No serious procedure-related complications Ascites: 21%	HE improvement: 100%
Philips et al, 2017	21	Splenorenal-17 Mesocaval-7 Rest-other types	CARTO, PARTO, SSO; 95.2%	1–9 months	1 mortality-hemoperitoneum EV : no significant increase GIB: 1 nonfatal, ascites: no significant increase	HE improvement: Short-term follow-up: 71% Long-term: 23%
Philips et al, 2020	45	Splenorenal-25 Mesocaval-4 Paraumbilical-4 Rest-other types	BRTO, CARTO, PARTO, CAATO; 100%	18 months	Two study groups-early (first episode of HE) and late (recurrent HE) shunt embolization Ascites, GIB, recurrence of HE, PVT lower in early shunt embolization group	Recurrence of HE%-4.5 in the early embolization group versus 28.6% in the late embolization group, at 9 months

BRTO: balloon-occluded retrograde transvenous obliteration, CARTO: coil-assisted retrograde transvenous obliteration, PARTO: plug-assisted retrograde transvenous obliteration, SSO: surgical shunt occlusion, CAATO: coil-assisted antegrade transvenous obliteration, EV: esophageal varices, GIB: gastrointestinal bleeding, HE: hepatic encephalopathy, and PVT: portal vein thrombosis.

**Table 2:** Overview of recent studies on efficacy and safety of portosystemic shunt embolization for gastric variceal bleeding.

Authors, year	*n*	Technique; success rate	Follow-up	Complications	Comments
Sabri et al, 2014	50	BRTO: 91%	18.2 months (mean)	9% (2 of 23); hospital-acquired pneumonia, pulmonary embolism (treated medically).	Study comparing BRTO and TIPS for GV. No recurrence of GV bleed in the BRTO group; 11% in TIPS group. HE in 15% of TIPS group; none in BRTO group.
Kim et al, 2016	95	BRTO, PARTO; 94.7%	12 months (mean)	Hemoglobinuria in 1 patient and death in one patient due to DIC in the BRTO-EO group. No major complications in the other 2 groups.	Less complications with BRTO using STS foam or PARTO compared to BRTO using EO. Recurrence more common with PARTO. Shortest procedure time with PARTO.
Chang et al, 2016	19	PARTO; 94.7%	11 months (median)	7 minor complications (fever, hypotension, microscopic hematuria). EV (new onset or aggravated) in 5 patients, one died at 7 months due to EV bleed.	No recurrence of GV bleed in any patient.
Kim et al, 2017	52	BRTO; 88%	727 days (mean)	Balloon rupture in 2 patients, common femoral artery injury requiring arteriotomy in 1 patient.	Study comparing BRTO and TIPS for GV. No significant difference in procedural complications, rebleeding rates, new onset ascites or mean survival between the two groups. HE more common in TIPS group.
Lee et al, 2017	142	BRTO; 86.2%	28.2 months (mean)	Pulmonary edema in 1 patient (recovered). HE in 30% of patients in TIPS group; none in BRTO group. Exacerbation of ascites in 14% of patients in BRTO group; 4% in TIPS group.	Study comparing BRTO and TIPS for GV. Lower rebleeding rates and better overall postprocedure survival rates after BRTO.
Gimm et al, 2018	176	BRTO; 95.7%	NA	No difference in procedural complications, aggravation of ascites, EV, pleural effusion, HE. Progression of ascites higher in BRTO group.	Study comparing BRTO and TIPS for GV. Better overall survival and rebleeding-free survival with BRTO.

BRTO: balloon-occluded retrograde transvenous obliteration, PARTO: plug-assisted retrograde transvenous obliteration, EV: esophageal varices, GV: gastric varices, HE: hepatic encephalopathy, STS: sodium tetradecyl sulfate, EO: ethanolamine oleate, DIC: disseminated intravascular coagulation, TIPS: transjugular intrahepatic portosystemic shunt, and NA: not applicable.

## Data Availability

Data regarding this study are available on request to the corresponding author.
